# Volumetric-modulated arc therapy for left-sided breast cancer and all regional nodes improves target volumes coverage and reduces treatment time and doses to the heart and left coronary artery, compared with a field-in-field technique

**DOI:** 10.1093/jrr/rrv052

**Published:** 2015-09-19

**Authors:** Marguerite Tyran, Hugues Mailleux, Agnes Tallet, Pierre Fau, Laurence Gonzague, Mathieu Minsat, Laurence Moureau-Zabotto, Michel Resbeut

**Affiliations:** Service de Radiothérapie, Institut Paoli-Calmettes, 232, boulevard Sainte-Marguerite, 13009 Marseille, France

**Keywords:** breast cancer, VMAT, field-in-field, left coronary artery

## Abstract

We compared two intensity-modulated radiotherapy techniques for left-sided breast treatment, involving lymph node irradiation including the internal mammary chain. Inverse planned arc-therapy (VMAT) was compared with a forward-planned multi-segment technique with a mono-isocenter (MONOISO). Ten files were planned per technique, delivering a 50-Gy dose to the breast and 46.95 Gy to nodes, within 25 fractions. Comparative endpoints were planning target volume (PTV) coverage, dose to surrounding structures, and treatment delivery time. PTV coverage, homogeneity and conformality were better for two arc VMAT plans; V95%^PTV-T^ was 96% for VMAT vs 89.2% for MONOISO. Homogeneity index (HI)^PTV-T^ was 0.1 and HI^PTV-N^ was 0.1 for VMAT vs 0.6 and 0.5 for MONOISO. Treatment delivery time was reduced by a factor of two using VMAT relative to MONOISO (84 s vs 180 s). High doses to organs at risk were reduced (V30^left lung^ = 14% using VMAT vs 24.4% with MONOISO; dose to 2% of the volume (D2%)^heart^ = 26.1 Gy vs 32 Gy), especially to the left coronary artery (LCA) (D2%^LCA^ = 34.4 Gy vs 40.3 Gy). However, VMAT delivered low doses to a larger volume, including contralateral organs (mean dose [Dmean]^right lung^ = 4 Gy and Dmean^right breast^ = 3.2 Gy). These were better protected using MONOISO plans (Dmean^right lung^ = 0.8 Gy and Dmean^right breast^ = 0.4 Gy). VMAT improved PTV coverage and dose homogeneity, but clinical benefits remain unclear. Decreased dose exposure to the LCA may be clinically relevant. VMAT could be used for complex treatments that are difficult with conventional techniques. Patient age should be considered because of uncertainties concerning secondary malignancies.

## INTRODUCTION

Adjuvant radiotherapy (RT) for breast cancer is a standard treatment used to improve local tumor control and overall survival [1–4]. Lymph node irradiation is used as an additional treatment for high-risk patients. However, these large treatment volumes lead to coverage difficulties, with junction issues between the breast and node fields and exposure of organs at risk (OARs) to higher radiation doses. Some groups include the internal mammary chain (IMC) in the tangential fields. This technique is called the modified wide tangent (MWT) [5]. The use of intensity-modulated radiation therapy (IMRT) allows a homogeneous dose distribution in many kinds of treatments. Volumetric-modulated arc therapy (VMAT) for left-sided breast treatment, including lymph node irradiation involving the IMC, was recently compared with MWT [6, 7], but never with a forward-planned multi-segment technique with a mono-isocenter (MONOISO). Target volumes coverage appeared better with VMAT than with MWT. The goal of our study was to compare the dosimetric results of two IMRT techniques in this treatment subset: VMAT vs MONOISO, in order to assess whether or not there is an advantage for VMAT.

## MATERIALS AND METHODS

### Patient selection

We performed 10 planning studies for patients with left-sided breast cancer, eight after breast conservative surgery and two after mastectomy. Diverse anatomies were selected. Using the same contours and scanner datasets, treatment planning was provided by Pinnacle® (version 9.2) for MONOISO and by Raystation® (version 4.0) for VMAT. Treatment planning was performed using an Elekta multileaf collimator (MLC) with a 5-mm leaf width (Agility, Elekta, UK).

The clinical target volume (CTV) included the breast/chest wall (CTV^T^), and the supraclavicular axillary level II and III nodes and the IMC in the first three interspaces (CTV^N^). The CTV was extended by 5 mm circumferentially, creating the planning target volume (PTV^outside^), both for breast (PTV^T^ outside) and for nodes (PTV^N^ outside). For planning evaluation, the PTV outside was restricted to 5 mm under the skin and was referred as the PTV. The breast/chest PTV volumes ranged from 256 to 1458 cm^3^. The prescription dose to the breast/chest wall was 50 Gy in 25 fractions (f). The nodal volume received 46.95 Gy in 25 f (corresponding to 46 Gy in 23 f with α/β = 4). We did not take into account the tumor bed treatment. The OARs (lungs, heart, left coronary artery [LCA], esophagus, humeral head, thyroid and right breast) were also contoured, according to the RTOG recommendations [8].

### Forward-planned multisegment and monoisocentric technique

**Fig. 1.**
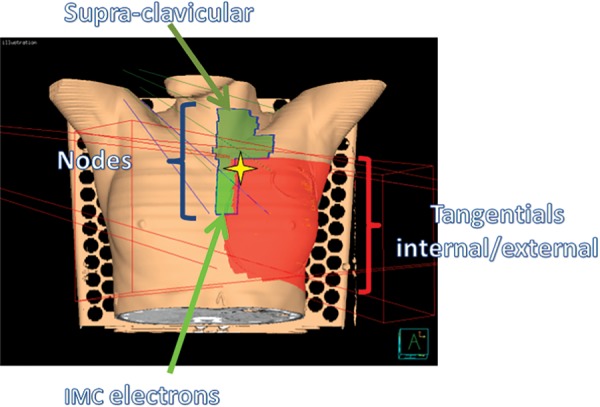
Fields description of the forward-planned multisegment technique with a mono-isocenter: tangential fields (constructed from PTV^outside^ +5mm right/superior/posterior, 15mm left/anterior and 10mm inferior, with a 7mm maximum overlap with the node field), node field (+5mm in every direction, with the exception of antero–posterior), IMC field (inferior region of the node field, 3cm wide and 15°angle), supra-clavicular field (superior region of the node field) and axillar post field to cover the external region of the PTV^N^).

Tangential and node fields were constructed from the PTV^outside^ with margins. In order to avoid an underdosage, an overlap of ≤7 mm between the tangential and node fields was accepted. We used 6-MV photons (or a mixture of 18- and 6-MV photons for large volumes). The IMC field was treated using a combination of photons and electrons. The upper limit of this field was chosen so that the CTV was at a constant depth and could be treated with same electron energy. That depth defined the position of the unique isocenter that was also the superior corner of the IMC electron field. Contralateral OARs were excluded (Fig. 1).

The field-in-field optimization used segments provided by the MLC and consisting of a few monitor units (MUs) in the main tangential field to erase areas of overdose by increments of 6% [9]. Three or four segments were usually used; the main segment that corresponded to the whole tangential field consisted of ∼80% of the MU. Dose calculation was carried out according to a collapse-cone algorithm for photons and pencil beam from Hogstrom [10] for electrons, on a 3 × 3 mm matrix.

### VMAT

For arc-therapy plans, we exclusively used the Elekta system with 6-MV photons. Our clinical goals were in accordance with the external RT guidelines published in 2007 [11].

We kept a significant proportional difference between the objectives of target volumes (weight = 100) and those of OAR (weight = 1). We used 13 objectives, requiring a uniform dose of 50 Gy, a minimum dose (Dmin) of 49 Gy and a maximum dose (Dmax) of 51 Gy on the PTV^T−outside^; and a uniform dose of 46.95 Gy, a minimal dose of 46 Gy and a maximum dose of 48 Gy on PTV^N−outside^. We included two additional objectives to achieve improved conformality: the planning system was required not to exceed 47.5 Gy (i.e. 95% of the prescribed dose) to a ring that was constructed by adding 10 mm around the PTVs, and not to exceed 25 Gy to a control zone that was defined as all healthy tissues outside the PTVs and rings. For the left lung, the objective was to give <15 Gy to 20% of its volume, and for the heart <10 Gy to 10% of its volume. For the LCA, an objective of 25 Gy for the Dmax was retained [12]. For the contralateral organs (lung and breast) we used an identical objective of 3 Gy to 20% of the volume. A valid plan should respect the prescription regarding the PTV^T^ and fulfill the clinical goals. These clinical goals were defined as follows: 30 Gy to 5% of the heart; V20 <30% and V30 <20% for the left lung; and mean dose (Dmean) <4 Gy for contralateral organs (lung and breast). In addition, the healthy tissues should not receive >55 Gy to 0.1 cm^3^. For target volumes, 96% of their volume should be covered by 95% of the prescribed dose. We used two arcs from 290° to 170° clockwise and inverse, with one control point every 4°.

**Fig. 2.**
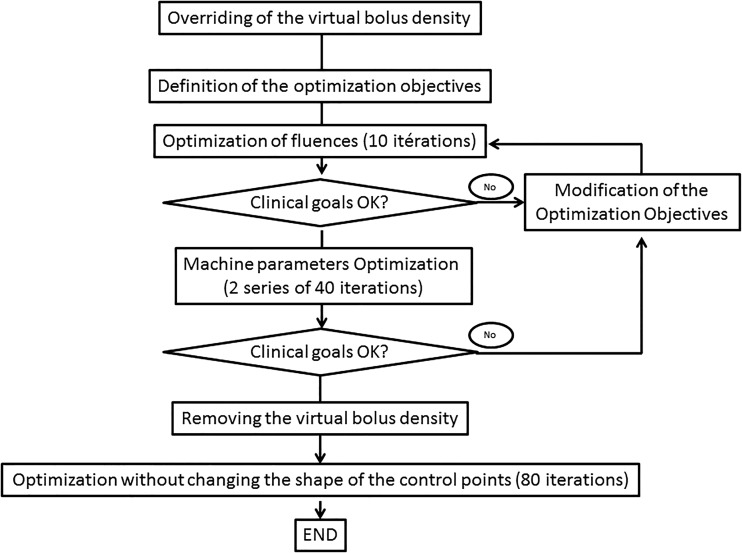
Flow diagram showing the volumetric-modulated arc therapy planning process.

The optimization process is shown in Fig. 2. To anticipate breast anatomical modification and lack of breast tissue coverage during treatment, we used a virtual bolus during the first optimization. A similar approach was described by Nicolini *et al.* [13]. This bolus was removed when the clinical goals had been achieved. Then, we restarted the process without changing the shape of the segment.

### Comparison criteria

Target volume coverage should be in conformity with the ICRU83 [14]. The homogeneity index (HI) was calculated according to the following formula: HI = D_2%_ – D_98%_/D_50%_, where D_2%_, D_98%_ and D_50%_ = dose to 2%, 50% and 98% of the volume, respectively. The conformity index (CI) regarding the combined PTV was calculated according to Van't Riet *et al*. [15] as follows: CI = TV_RI_/TV × TV_RI_/VRI (TV: Target Volume, TV_RI:_ Target volume covered by the prescription isodose, VRI: volume of the prescription isodose). We also evaluated the number of MUs and the treatment time. Treatment time didn't take into account set-up time and imagery control time. All results were analyzed using a Wilcoxon's signed rank test and considered significant if *P* was <0.05. The study was approved by the institutional review boards of our institution.

## RESULTS

**Fig. 3.**
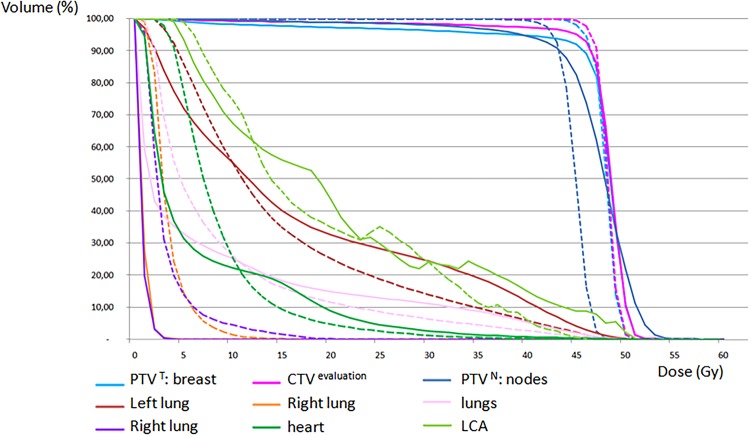
Dose–volume histograms. MONOISO (solid lines) and VMAT (dashed lines). PTV = Planning Target Volume; CTV = Clinical Target Volume; LCA = Left Coronary Artery.

Dose distributions for target volumes and OARs are summarized in Table 1 and Fig. 3.

**Table 1.** Plan comparison parameters, mean values and range for VMAT and MONOISO for this study

	MONOISO			VMAT			Wilcoxon's signed rank test
	Mean	min	max	Mean	min	max	
**PTV^T^: breast**							
D2% (Gy)	**53.0**	52.5	54.1	**52.2**	51.7	52.7	0.004
Dmean (Gy)	**49.2**	44.8	50.5	**49.9**	49.9	50	0.188*
Dmedian (Gy)	**50.8**	50.2	51.2	**50.0**	49.9	50	0.002
V95% (%)	**89.2**	67.6	94.3	**96.0**	94.9	97.6	0.002
HI	**0.6**	0.2	0.9	**0.1**	0.1	0.1	0.002
**CTV^evaluation^**							
V95% (%)	**94.1**	82	97.5	**98.9**	98.6	99.6	0.002
**PTV^N^: nodes**							
V95% (%)	**86.7**	73.5	93.3	**96.0**	95.2	97	0.002
D2% (Gy)	**52.1**	51	53.9	**49.1**	48.9	49.3	0.002
Dmedian (Gy)	**48.1**	46.4	49.6	**47.0**	46.9	47	0.008
HI	**0.5**	0.2	0.9	**0.1**	0.1	0.1	0.002
**PTV^combined^** CI	**0.6**	0.4	0.7	**0.8**	0.7	0.8	0.063*
**Heart**							
Dmean (Gy)	**6.7**	4.2	9.6	**8.6**	6.6	10.2	0.063*
D2% (Gy)	**32.0**	25.2	38.5	**26.1**	19.3	32.8	0.002
V30 (%)	**2.7**	1.1	5.2	**1.3**	0.2	2.9	0.006
**LCA**							
Dmean (Gy)	**19.5**	11.1	31.3	**18.1**	11.1	26.3	0.492*
D2% (Gy)	**40.3**	22.3	50	**34.4**	18.8	44.7	0.018
**Left lung**							
Dmean (Gy)	**16.9**	13.6	20.2	**15.2**	14	16.7	0.025
D2% (Gy)	**46.9**	45.2	48.8	**44.9**	42.7	47.2	0.01
V30 (%)	**24.4**	19.2	31.6	**14.0**	11.3	17.1	0.002
V20 (%)	**32.7**	26	41.2	**25.4**	23	28.8	0.002
V10 (%)	**55.1**	42.9	67.1	**55.3**	43	63.3	0.77*
V5 (%)	**72.1**	57.2	84.6	**86.7**	78.5	97.2	0.004
**Right lung**							
Dmean (Gy)	**0.8**	0.6	1	**4.0**	3	8.5	0.002
D2% (Gy)	**2.2**	1.7	2.7	**9.3**	7.9	10.4	0.002
V10 (%)	**0.1**	0	0.3	**7.4**	0.6	59.6	0.002
V5Gy (%)	**0.2**	0	0.6	**14.5**	11.6	18.2	0.002
**Lungs**							
Dmean (Gy)	**8.1**	6.5	9.8	**8.8**	7.7	9.6	0.016
V5Gy (%)	**33.0**	26	37.8	**47.3**	39.9	54.1	0.002
**Right breast**							
Dmean (Gy)	**0.4**	0.3	0.6	**3.2**	2.5	3.7	0.002
D2% (Gy)	**6.7**	1	52.9	**12.3**	6.3	17.8	0.084*
V5Gy (cm^3^)	**0.0**	0	0	**90.0**	27.7	147	0.002
V5Gy (%)	**0.0**	0	0	**13.8**	8	17.5	0.002
**Humeral head**							
Dmean (Gy)	**3.7**	2.1	7.3	**10.4**	5.8	14.8	0.002
D2% (Gy)	**17.8**	4.3	35.7	**25.1**	20.0	28.6	0.084*
V50Gy (%)	**0.0**	0.0	0.0	**0.0**	0.0	0.0	
V30Gy (%)	**1.7**	0.0	7.4	**0.2**	0.0	1.0	0.125*
V20Gy (%)	**3.0**	0.0	11.8	**11.0**	2.0	26.0	0.002
**Thyroid**							
vol (cm^3^)	**12.0**	4.2	40.0				
Dmean (Gy)	**24.5**	15.6	31.2	**31.1**	22.4	34.3	0.002
D2% (Gy)	**51.8**	48.5	53.6	**48.3**	47.8	48.7	0.004
V30Gy (%)	**45.6**	24.8	61.1	**46.8**	23.8	64.6	0.125*
V35Gy (%)	**45.1**	24.1	59.6	**44.5**	19.8	57.4	0.438*
V40Gy (%)	**44.1**	23.2	56.5	**42.0**	17.4	51.1	0.109*
Dmean (Gy) left lobe	**49.0**	42.7	51.8	**45.3**	42.0	46.9	0.008
Dmean (Gy) right lobe	**4.0**	2.6	7.5	**19.3**	14.6	22.9	0.002
**Esophagus**							
Dmean (Gy)	**7.2**	5.9	9.0	**11.9**	10.2	14.7	0.002
D2% (Gy)	**41.5**	27.9	45.6	**41.5**	30.8	46.1	0.695*
V20Gy (%)	**11.6**	6.4	18.0	**18.2**	10.5	23.5	0.002
V45Gy (%)	**1.5**	0.0	8.0	**1.6**	0.0	7.4	0.625*
**MU**	**405**	381	420	**430**	376	480	0.02
**Treatment time (s)**	**180**	nr	nr	**84**	81	86	

* *P* < 0.05, according to the Wilcoxon's signed rank test. Min = minimum; max = maximum; PTV = Planning Target Volume; CTV = Clinical Target Volume; LCA = Left Coronary Artery; D2% = dose to 2% of the volume; Vn(%) = percentage volume receiving ≥nGy; Dmean = mean dose; Dmedian = median dose; HI = homogeneity index; CI = conformity index; MU = monitor unit.

**Fig. 4.**
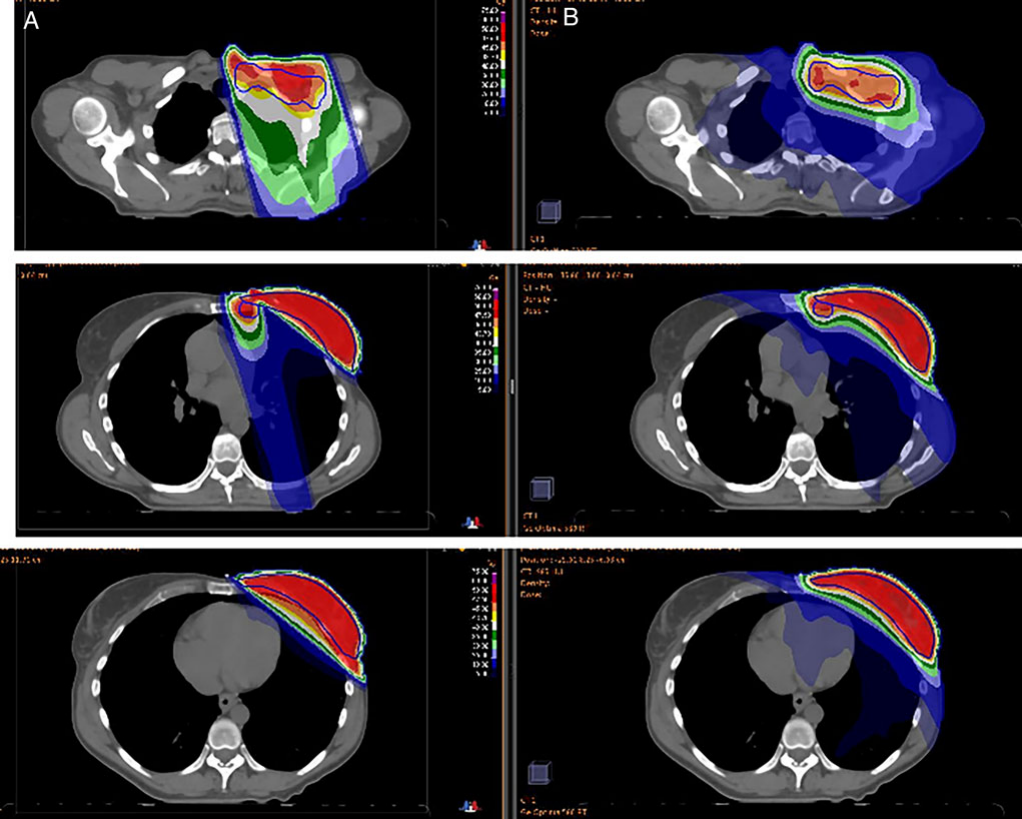
Dose distribution of a 3D conformal radiotherapy with field-in-field (**A**) and two-arcs (**B**): PTVT and N (black), and isodose lines of 5 Gy (dark blue), 10 Gy (blue), 25 Gy (light blue), 30 Gy (light green), 35 Gy (dark green), 40 Gy (white), 43.7 Gy (yellow), 45 Gy (orange), 47.5 Gy (dark red) and 50 Gy (red).

PTV coverage, homogeneity and conformality were better for two arc VMAT plans; V95%^PTV-T^ was 96% (94.9–97.6) for VMAT vs 89.2% (67.6–94.3) for MONOISO (*P* = 0.002)*.* HI^PTV-T^ was 0.1 (0.09–0.13) and HI^PTV-N^ was 0.1 (0.1–0.12) for VMAT vs 0.6 (0.18–0.92) and 0.5 (0.17–0.88) for MONOISO (*P* =0.02). Treatment delivery time was reduced by a factor of two using VMAT relative to MONOISO (84 s vs 180 s). High doses to organs at risk were reduced (V30^left lung^ = 14% [11.3–17.1] using VMAT vs 24.4% [19.2–31.6] using MONOISO, *P* = 0.02; D2%^heart^ = 26.1 Gy [19.3–32.8] vs 32 Gy [25.2–38.5] *P* = 0.02), especially to the LCA (D2%^LCA^ = 34.4 Gy [18.8–44.7] vs 40.3 Gy [22.3–50.0] *P* = 0.018). However, VMAT delivered low doses to a larger volume, including contralateral organs (Dmean^right lung^ = 4 Gy [3–8.5] and Dmean^right breast^ = 3.2 Gy [2.5–3.7]). These were better protected using MONOISO plans (Dmean^right lung^ = 0.8 Gy [0.6–1] and Dmean^right breast^ = 0.4 Gy [0.3–0.6] *P* = 0.02). Mean doses to the humeral head, thyroid and esophagus were also higher for VMAT plans (Dmean^humeral head^ = 10.4 Gy [5.8–14.8], Dmean^thyroid^= 31.1 Gy [22.5–34.3] and Dmean^esophagus^ = 11.9 Gy [10.2–14.7] vs 3.7 Gy [2.1–7.3], 24.5 Gy [15.6–31.2] and 7.2 Gy [5.9–9], respectively, for MONOISO.

## DISCUSSION

We chose to study one of the worst case scenarios with respect to complexity of treatment volume, namely the left breast with whole node irradiation. Because of the limited number of patients involved, it was not possible to conduct a statistical analysis. Nevertheless, we selected a large variety of chest anatomies, allowing for the application of our VMAT technique in most cases. It should be noted that there have been only two previous studies [6, 7] concerning this subject, and they analyzed only five cases, as opposed to the 10 cases evaluated in our study. It is worth noting that in the two published studies [6, 7], the dose to the tumor bed wasn't taken into account, as has been done in this work.

Only the VMAT plans achieved delivery of 95% of the prescribed dose to 95% of the PTVs and respected median doses to both PTVs (Table 1). Median doses delivered to the target volumes using MONOISO were higher than the prescribed doses. These results reflect the advantages regarding the homogeneity and conformity of the VMAT plans, as suggested by the improved HI and CI.

The high doses delivered to the heart and the LCA (illustrated by the D2%) were reduced using the VMAT plans. The mean dose to the heart was acceptable using the VMAT plans, but this was even lower using a forward-planned multisegment technique with a mono-isocenter. The left lung received a comparable V10 using both plans; however, it received an inferior mean dose, D2%, V30 and V20, and a higher V5 using the VMAT plans. Doses to contralateral organs were low using VMAT treatment planning, but still higher than using MONOISO. It should be noted that the average V5 covered 90 cm^3^ of the right breast volume using the VMAT plans, whereas it was completely excluded from this isodose using the MONOISO plans.

The number of MUs was comparable in both plans. Treatment time was reduced by a factor of two using the VMAT as compared with the MONOISO plans, and by a factor of four if the efficiency of delivery was taken into consideration. From our recent experience, shorter treatment planning and delivery times are attractive with respect to workload (Fig. 4).

Comparable studies with ours used MWT as the reference arm [6, 7]. They demonstrated a benefit for VMAT treatment planning. However, results from the use of MONOISO as compared with MWT plans in our study indicated that they are superior, especially regarding OARs, as displayed in Table 2. For example, MW delivered higher doses to the heart and LCA, located in the wide tangential fields. For this reason, the question was asked of whether the advantages of VMAT over MWT were still accurate when compared with a forward-planned multisegment technique with a mono-isocenter.

**Table 2.** Plan comparison parameters, mean values for VMAT and MONOISO for this study and for other studies of the literature concerning similar volumes treated with static and dynamic intensity-modulated radiotherapy

			VMAT (study)	MONO ISO (study)	Popescu [6]		Sakumi [7]		Pasler [16]	Caudrelier [24]	Goddu [23]	Jagsi [31]	Krueger [25]	Dogan [29]	Van der Laan [28]	Beckham [27]	Popescu [26]	Cozzi [30]
*n* =			10		5		5		10	10	10	10	10	10	10	30	5	10
Technique			VMAT	MONO ISO	Rapid Arc	MWT	VMAT	MWT	VMAT	TOMO	TOMO	IMRT 9 fields	IMRT 9 fields	IMRT 9 fields	IMRT 9 fields	IMRT 11 fields	IMRT 11 fields	IMRT
**Target volumes**			Left breast supra-clav, II III, IMC		Left breast supra-clav, II III, IMC		Left breast supra-clav, III IMC		Left breast supra-clav, III + tumor bed	Left breast supra-clav, II III, IMC	Left breast supra-clav, II III, IMC	Left chest-wall supra-clav, III, IMC	Left chest-wall supra-clav, II III, IMC	Left chest-wall supra-clav, I II III, IMC	Left chest-wall supra-clav, IMC	Left breast, IMC	Left breast, IMC	Left and right breasts, IMC
**Dose PTV^T^**			50		50		50		50 + 10	50	50	60.0	50	50	50	50		48.6–50.0
**PTV^N^**			47		45		45		50	50	50	52.2	50	50	50	50		
**PTV^T^: breast**																		
Dmean (Gy)			50.0	49.0			52.0		51.7^c^		52.0^c^	50.2						
D2% (Gy)			53.0	52.0	51.8	54.3	56.0		55.9^c^			61.3^d^		56.0				
V95% (%)			96.0	89.0	95.0	92.0	95.0		35.4 (D98%)^c^	99.0^c^	98.0^c^	96.7	78.0^e^	50Gy (D95%)	98.5			86.7
HI			0.1	0.6	0.9^a^	0.8^a^	2.1	1.9		1.13^a^						0.95^a^	0.96^a^	
**CTV ^evaluation^**																		
**V95% (%)**			99.0	94.0														
**PTV^N^: nodes**																		
V95% (%)		96.0		86.7	99.0	95.3	98.7^b^					53.5		50.6 (D95%)	99.3			
D2% (Gy)		49.0		52.0	49.0	54.0						57.4^d^		55.1				
HI		0.1		0.5			0.4		0.4^c^	0.69^c^								
**CI** **Combined PTV**	0.8			0.6	0.7	0.4			0.8							0.9	0.9	1.2
**UM**		430		405	862	489												
**Treatment** **time (s)**		84		180	240	300												
**Heart**																		
V30 (%)		1.3		2.7	2.6	16.0	3.0	14.0	2.7	1.5		0.0	0.1		5.3	0.7	3.0	
D2% (Gy)		26.0		32.0					32.0							47.0		53^f^
Dmean (Gy)		8.6		6.7	11.0	11.0	11.0		8.9	7.0	12.0	7.2		4.1	10.0		13.0	9.9
**LCA**																		
Dmean (Gy)		18.0		19.5								11.2						
D2%		34.4		40.3								19.3^d^						
**Left lung**																		
Dmean (Gy)		15.0		17.0	12.0	18.0	13.0		15.0	8.3	12.0	18.0	9.5	6.9			12.0	16.0
D2% (Gy)		45.0		47.0					50.0					43.0				
V30 (%)		14.0		24.0					17.0	5.1	8.9	17.8	0^e^	10.0				19.0
V20 (%)		25.0		33.0	17.0	37.0	19.0	38.0	27.0	9.2	18.0	34.4	8^e^	20.0		17.0	15.0	29.0
V10 (%)		55.0		55.0	40.0	41.0					35.0	67.8	20^e^			47.0		
V5 (%)		87.0		72.0	70.0	47.0					74.0	93.4	52^e^			84.0		
**Right lung**																		
Dmean (Gy)		4.0		0.8	2.9	1.0	4.0		3.4	6.2	4.2	3.2	5.8			4.0	4.0	6.3
D2% (Gy)		9.3		2.2					11.0					8.5				27.5^f^
V5 (%)		15.0		0.2	8.1	0.0	22.0		23.0	38.0	26.0	11.1				14.0	14.0	49.0
**Lungs**																		
V5Gy (%)		47.0		33.0							47.0				45.0			
**Right breast**																		
Dmean (Gy)		3.2		0.4	3.2	4.0	3.1		2.8	4.8	4.3	2.7	2.8		2.7	4.0	4.0	4.1
D2% (Gy)														3.6				23^f^
V5 (cm^3^)		90.0		0.0	52.0	85.0	40.0					10.6^g^			9^g^	29^g^	32^g^	34^g^

^a^HI calculated with following formula = D2%/Dr; ^b^V95% for supra-clavicular area alone; ^c^Data concerning PTV-T combined to PTV-N; ^d^D1% (Gy); ^e^ = data derived from figures; ^f^Dmax; ^g^V5(%); min = minimum; max = maximum; PTV = Planning Target Volume; CTV = Clinical Target Volume; LCA = Left Coronary Artery; D2% = dose to 2% of the volume; Vn(%) = percentage volume receiving ≥*n* Gy; Dmean = mean dose; Dmed = median dose; HI = homogeneity index; CI = conformity index; MU = monitor unit; MWT = Modified Wide Tangent; TOMO = tomotherapy; IMRT = Intensity Modulated Radiotherapy; supra-clav = supra-clavicular; IMC = internal mammary chain.

Other studies concerning arc therapy for breast cancer have reported comparable results; however, Pasler *et al*. [16], Subramaniam *et al*. [17], Johansen *et al.* [18] and Badakhsi *et al*. [19] mixed the results for right and left breasts in their studies and excluded the IMC. Yong-Yin *et al*. [20], Guang-Hua Jin *et al*. [21] and Tsai *et al*. [22] considered the left breast only, but did not undertake node irradiation. We also compared our results with those of Goddu *et al*. [23] and Caudrelier *et al*. [24] concerning tomotherapy. The results of eight studies involving static IMRT [25–30] are also included in Table 2, with gaps in data varying according to the publications. Indeed, though the dose to the PTV-T is almost always reported, some other dosimetric data are missing. Data are more readily available for OARs.

Our VMAT results are in accordance with those of Popescu *et al*. [6] and Sakumi *et al*. [7]. It should be noted that Popescu *et al*. [6] excluded OARs from their PTV, which distorted their results. They also reported a reduced dose to the left lung, without specifying if they excluded the PTV from the lung. During the optimization process, we noticed an interdependency between doses to the heart and lung, which concur with their [6] finding of the delivery of a higher dose to the heart. However, their dose delivery to the right breast was superior, because they only looked at the dose to the medial contralateral breast.

Regarding the heart, Pasler *et al*. [16] reported inferior results to those of other authors, even though the IMC was not treated. Nevertheless, they realized that an overdose to the tumor bed could partly explain their data. Heart doses are strongly dependent on patient anatomy, making a quantitative comparison difficult. No study has reported the D2% to the LCA, which may be more relevant than the Dmean, as proposed by Jagsi *et al*. [31]. The lowest doses to the contralateral lung were reported by Popescu *et al*. [6]; other studies, especially Goddu *et al*. [23], have reported higher Dmean and V5 values, using tomotherapy.

Concerning VMAT, only planning studies without clinical results have been published to date. VMAT provides dosimetric advantages relative to traditional plans. These improvements are more visible when compared with MWT than compared with MONOISO, which is already a reliable technique that can be used in these complex situations.

The clinical implications of these dosimetric improvements remain unclear. Improved homogeneity has been proven to provide better cosmetic outcomes [32–34]. However, the benefit for local control and overall survival remains to be demonstrated. According to the older studies reviewed in the ECBTCG meta-analysis [4], the benefit of RT regarding overall survival after surgery is reduced as a result of non-cancer mortality, mostly (90%) linked to cardiovascular mortality; post-radiation coronary injury is the most studied area in this regard. Because of its location, the LCA is highly vulnerable when tangential fields are used. However, modern techniques, with MLC obscuring the heart and the use of mixed photons/electrons for IMC, have already reduced the level of non-breast cancer–related mortality [35]. A study by Darby *et al*. [36] described a linear relationship between the mean dose to the heart and cardiac events. There was no threshold under which no events occurred. These authors failed to demonstrate a relationship between the mean dose to the LCA and cardiac events, because of the difficulty in visualizing that structure. However, this issue has to be taken into account and in our study, despite a higher mean heart dose with VMAT, the maximum dose to the heart and to the LCA were decreased. Other groups have studied the doses to the LCA. The LCA is a ‘serie organ’; thus, its constraint should be the Dmax. Untereiner *et al*. [12] reported a Dmax of 22.94 Gy and a mean dose of 15.84 Gy. Fenoglietto *et al*. [37] reported a D1% of 29.2 Gy and a mean dose of 14.1 Gy. Our VMAT plans significantly reduced the D2% to the LCA. The benefit of lowering high doses to the heart and the LCA may be clinically relevant for reducing cardiac toxicity.

Another option for reducing cardiac radiation exposure involves techniques that keep the heart outside the radiation fields (breathing control, maintaining patients in the prone position and partial RT). These techniques have demonstrated dosimetric improvements [38], but their clinical benefit regarding arc therapy has still to be evaluated. The impact of systemic treatment, such as other cardiovascular risk factors, should also be taken into consideration.

Data concerning secondary cancers that occur after breast RT are poor. The ECBTCG meta-analysis [4] reported an increased number of contralateral breast (*P* = 0.002) and lung (*P* = 0.0007) cancers after high-dose exposures near target volumes. Berrington de Gonzalez *et al*. [39] published data on an important series of surviving patients; 9% developed secondary cancer, and only 8% of these were caused by RT. The risk was higher for patients aged <45 years at the time of treatment. Regarding secondary lung cancer, a recent study [40] demonstrated a linear increase in risk. The risk was even greater for patients who smoked. Two studies [41, 42] concluded that the risk of developing contralateral breast secondary cancer was higher for patients aged <40 years at the time of treatment. Stovall *et al*. [41] carried out one of the few studies that have presented findings according to the location of contralateral breast secondary cancer. There were more cases in external quarters and the risk was dose dependent [41].

New RT techniques have given rise to questions regarding the impact of low-dose exposure on carcinogenesis. Some modeling has suggested that there is a doubling of the risk using IMRT [43, 44]. These low doses are caused by many factors, including scattered dose in the patient, from the head of the accelerator (collimator, leafs and blocks), and radiation leakage from the head. This leakage may be lower for IMRT than for conventional 3D treatment. It has been shown [45] that the accuracy of the convolution–superposition algorithm is limited in the out-of-field region, which can lead to underestimated doses outside the field. For VMAT, the estimated dose is more reliable, because it is partly the result of the treatment field itself [46]. Most modeling regarding second cancer risk uses the linear–exponential risk model, but other models [47, 48] may be more compatible with clinical practice. Even if there are uncertainties concerning its quantification, and despite the better dose distribution of VMAT than a forward-planned multi-segment technique with a mono-isocenter, we cannot neglect the theoretically increased risk of secondary cancers using new RT techniques. Apart from organizational concerns, the choice of the irradiation technique should be guided by many criteria such as patient age, tumor stage and localization, patient anatomy and comorbidities. Many parameters can be modulated to optimize the differential effect on tumor/healthy tissues, including: radiobiological parameters (volume, dose, staging and fractionation); technical parameters (IMRT, breathing control and radiation energy); and individual parameters such as patient and tumor sensitivity to radiation [49].

VMAT improved PTV coverage and dose homogeneity, but clinical benefits remain unclear. Decreased dose exposure of the LCA may be clinically relevant. VMAT could be used for complex treatments difficult with conventional techniques. Patient age should be considered because of uncertainties concerning secondary malignancies.

## FUNDING

Funding to pay the Open Access publication charges for this article was provided by Institut Paoli Calmettes.
